# Design of a Reconfigurable Crop Scouting Vehicle for Row Crop Navigation: A Proof-of-Concept Study

**DOI:** 10.3390/s22166203

**Published:** 2022-08-18

**Authors:** Austin Schmitz, Chetan Badgujar, Hasib Mansur, Daniel Flippo, Brian McCornack, Ajay Sharda

**Affiliations:** 1 Biological and Agricultural Engineering, Kansas State University, Manhattan, KS 66502, USA; 2 Department of Entomology, Kansas State University, Manhattan, KS 66502, USA

**Keywords:** reconfigurable, agricultural robot, row crop navigation, crop scouting, pest control

## Abstract

Pest infestation causes significant crop damage during crop production, which reduces the crop yield in terms of quality and quantity. Accurate, precise, and timely information on pest infestation is a crucial aspect of integrated pest management practices. The current manual scouting methods are time-consuming and laborious, particularly for large fields. Therefore, a fleet of scouting vehicles is proposed to monitor and collect crop information at the sub-canopy level. These vehicles would traverse large fields and collect real-time information on pest type, concentration, and infestation level. In addition to this, the developed vehicle platform would assist in collecting information on soil moisture, nutrient deficiency, and disease severity during crop growth stages. This study established a proof-of-concept of a crop scouting vehicle that can navigate through the row crops. A reconfigurable ground vehicle (RGV) was designed and fabricated. The developed prototype was tested in the laboratory and an actual field environment. Moreover, the concept of corn row detection was established by utilizing an array of low-cost ultrasonic sensors. The RGV was successful in navigating through the corn field. The RGV’s reconfigurable characteristic provides the ability to move anywhere in the field without damaging the crops. This research shows the promise of using reconfigurable robots for row crop navigation for crop scouting and monitoring which could be modular and scalable, and can be mass-produced in quick time. A fleet of these RGVs would empower the farmers to make meaningful and timely decisions for their cropping system.

## 1. Introduction

The world’s population is rapidly increasing from 7.7 billion in 2022 to a projected 9.8 billion by the year 2050 [1]. To meet the demands of food, fuel, and fiber of a growing population with limited available resources (land and water) requires innovative, efficient, and sustainable approaches to increase the food production levels while maintaining the ecological balance. The sustainable increase in food production is significantly influenced by the crop input management practices (fertilizers and pesticides).

Many kinds of insects feed on crops, damaging plants and posing a serious threat to crop production. Approximately 45% of the annual food production is lost due to pest infestation [2]. Therefore, pesticides are extensively used in modern agriculture. Pesticide application is an effective and economical way to enhance crop yield quality and quantity, thus ensuring food security for the growing population around the globe. Approximately 2 million tonnes of pesticides are utilized worldwide annually. The United States is the major pesticide consumer in the world. Annually, 500 million kg of pesticides is used in the United States, which cost around USD 10 billion per year [3]. The United States accounts for approximately 16–18% of total world pesticide expenditure. Pesticides are a significant component of total farm production expenditures and are essential for farm budgeting and management. According to the USDA 2020 farm production expenditures report, the cost of agricultural chemicals was around USD 16.5 billion, which accounts for 4.5% of total farm expenditure [4]. In the United States, corn, soybean, cotton, wheat, and potato account for around 80% of the total pesticide use [5]. Corn is the top pesticide-consuming crop in the United States and receives about 39% of the total pesticides [5]. Soybean production has the next largest share, accounting for 22% of total pesticides applied [5,6]. The average pesticide cost for corn and soybean ranges from USD 57–84 per acre in the US [7,8].

In a nutshell, pest infestation causes significant crop damage during crop production; reducing crop yield and controlling pest is costly. Therefore, pest infestation should be minimized or controlled in the early growth stages, reducing the excessive application of pesticides. However, collecting accurate and timely information on pest infestation is a crucial aspect of integrated pest management. Moreover, several pests and diseases exhibit an uneven spatial distribution, with typical patch structures evolving around discrete foci (localized areas exhibiting symptoms), especially during the early stages of development [9,10,11].

Besides this, in today’s data-driven agriculture, there is a need to collect more crop information throughout the growing season to increase the cropping system’s output. A better understanding of soil moisture, nutrients, and pest concentrations allows farmers and researchers to develop crop management strategies and make informed decisions for cropping systems that will lead to healthier and more efficient crops, thus increasing production.

In recent years, Unmanned Aerial Vehicle (UAV) technology has allowed researchers and farmers to monitor large fields in a fast and efficient manner by flying over the crop [12,13,14,15,16]. However, scouting field crops such as corn, sorghum, and soybean under the canopy is much more challenging as the UAVs cannot see under the canopy after its closure. The sub-canopy region usually hosts a large number of insects and pest hotspots since it is always occluded and close to the ground surface. For example, the European corn borer lays eggs on the underside of leaves near the leaf midrib. Therefore, it is important to scout the sub-canopy region during crop growth [17]. Farmers usually perform manual scouting to locate, identify, and assess the pest infestation level, disease attacks, and nutrient deficiency. A current method for pest scouting involves collecting pest samples by hand to determine population density or observing plants for damage to the roots, stems, or leaves. Manual scouting is time-consuming and laborious. It also becomes very challenging to cover large fields and dense crop canopy. Particularly, the corn has a closed canopy (V10: ten leaves formed), and collecting information on the underside of the leaves is a problem. Therefore, an automated scouting vehicle is needed to collect sub-canopy-level crop information which would help farmers and researchers to make management decisions.

The recent advancement in technology has allowed the collection of crop information with the help of high-resolution cameras, remote sensing methods, and real-time information transfer to the farmers. This study aims to build a scouting vehicle that would carry a necessary sensor package to collect information at sub-canopy level within the corn field. The planned scout vehicle is a Reconfigurable Ground Vehicle (RGV) that can change its configuration to traverse between inter-row and intra-row, mainly for a corn crop. This reconfiguration characteristic of the vehicle provides the ability to move anywhere in the field without damaging the crops.

The developed RGV would serve as a mobile scout vehicle and could be an excellent alternative to current manual scouting methods. Fleets of small RGVs can be deployed to scout and monitor large fields. These vehicles will collect and share real-time information to the farm database, allowing farmers to make better management decisions. The RGV operating at the sub-canopy level would solve multiple issues related to crop monitoring. It would precisely locate the pest hotspot and allow targeted site-specific pesticide application, reducing pesticide application compared to the entire field application method. These savings significantly impact total production costs and would reduce the unsustainable chemical load on environments. The RGV fleet would empower the farmers and researchers to make meaningful and timely decisions.

The paper is organized into the following sections: Section 2 extensively reviews the currently available state-of-the-art scout vehicles and mobile robots. Section 3 describes the RGV design considerations for crop scouting operation, concepts of corn row detection, and the prototype RGV construction, fabrication, and control system. Section 4 discusses the laboratory and field test results of the RGV operation and power consumption. Section 5 includes the significant findings, concluding remarks, future scope, and research directions.

## 2. Review of Mobile Scout Vehicles

In the last few decades, several researchers have developed mobile robots or ground vehicles to perform agricultural operations ranging from tillage to harvesting in row crops or orchards. However, this paper solely discusses a few examples of crop scout vehicles.

**Figure 1:**
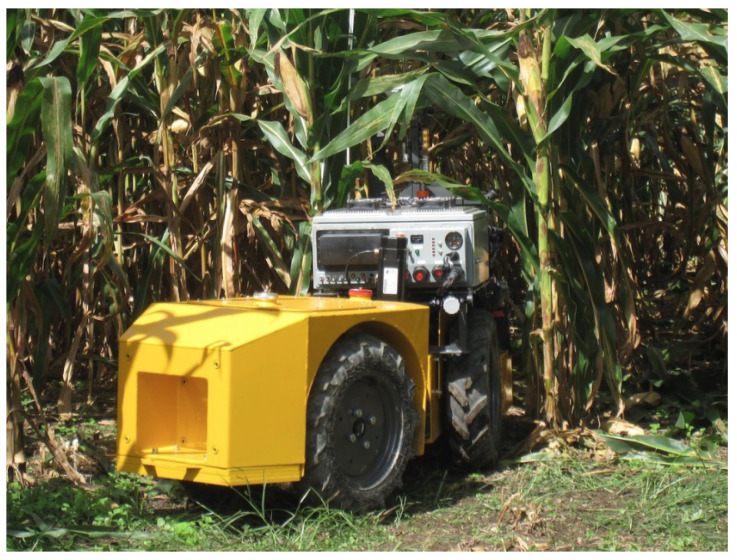
Rowbot entering a corn field [18].

The Rowbot (Figure 1) is a mobile robot designed to monitor and apply in-season nitrogen to corn. In addition, it is also used for inter-seeding the cover crops [18]. The Rowbot has a large turning radius and cannot operate in conventionally planted corn fields that have headlands without incurring some crop damage.

**Figure 2:**
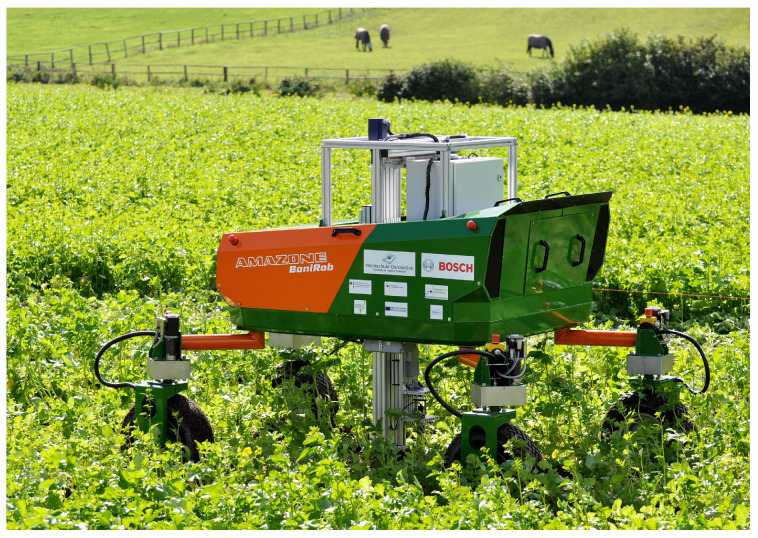
BoniRob collecting data in the field [19].

BoniRob (Figure 2) is another robotic platform for agricultural operation. It is a four-legged multi-row vehicle that straddles two rows of crops, collecting data as it drives down the rows [19]. The BoniRob can exchange different sensor packages for other management tasks. Similar to the Rowbot, BoniRob cannot traverse in a field with headlands and requires to run over crops.

**Figure 3:**
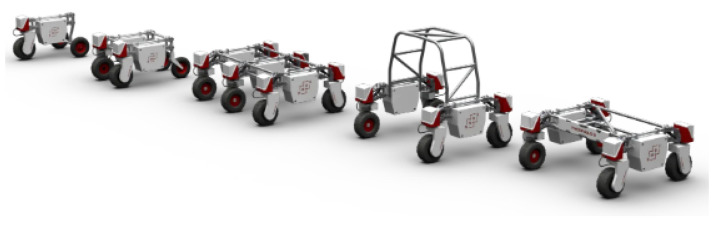
Thorvald, a modular agricultural robot [20].

Several other commercial robotic platforms have been designed for performing agricultural operations. Thorvald (Figure 3) is one example of a commercial automated platform (Saga Robotics Inc., Santa Maria, CA, USA). Thorvald is a modular system that performs various agricultural operations, including fruit and vegetable picking, weeding, spraying, crop phenotyping, transport, data collection, and monitoring. It is a lightweight vehicle with 180 kg and a payload of 250 kg. It is powered with a 48 V DC lithium-ion battery with a capacity of 70 Ah, which is enough for 10 h continuous operation, and also has a provision for battery replacement [20]. Thorvald is four-wheel steered but can be changed to two-wheel differential drive and achieves a maximum speed of 15 m/s. These specifications could vary depending upon their modular nature, as shown in Figure 3.

**Figure 4:**
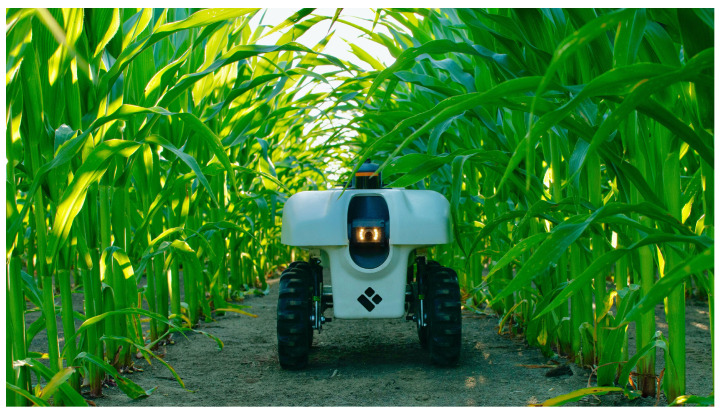
TerraSentia, a crop scout robot [21].

The University of Illinois and EarthSense Inc., Champaign, IL, USA developed a crop-phenotyping and scouting vehicle called TerraSentia (Figure 4). It is a small (330.2 mm), lightweight (10.8 kg) vehicle and can operate for 4 h with a single charge. TerraSentia is fitted with various sensors, including a camera, LiDAR, GPS, and other onboard sensors, to collect data on plant traits such as physiology and stress response [21]. It has been successfully used to collect corn and soybean field data. It is an ultra-compact robot, but it lacks the ability to travel intra-row.

AgBot II [22] is an autonomous prototype vehicle designed by the team of researchers and scientists from the Queensland University of Technology, Australia, for field scouting, site-specific operation, and weed management. The onboard sensor helps it to navigate a field, apply fertilizer, detect, classify, and kill weeds. More details on AgBot II can be found here [22]. Similarly, the University of Sydney, Australia, developed a solar-powered autonomous robot for field surveillance, mapping, and agricultural object classification and detection [23]. The prototype is fitted with multiple sensors, including LiDAR, thermal, infrared, hyperspectral cameras, and robotic manipulators used for mechanical weeding operations. To perform farm operations, the team also developed several successor prototypes, called ‘RIPPA’, ‘VIIPA’, ‘Mantis’, ‘Shrimp’, and ‘Digital FarmHand’.

Mobile robots or vehicles are also integral to greenhouse and nursery automation. HV-100 is a commercially available robot that performs in an unstructured, outdoor environment and was developed by Harvest Automation Inc., Billerica, MA, USA [24]. It is commonly used in greenhouse environment to perform repetitive, labor-intensive tasks. One such task is to move crop containers and it can handle 240 pots per hour under ideal conditions.

The literature survey suggests that a few prototype and commercial vehicles are available for row crop environments. However, to the best of our knowledge, there is a lack of information on autonomous scout vehicles. Many scout vehicle designs or configurations become a constraint for row crops planted with headland. The vehicles reviewed in this study performed very well in test plot and field planted straight to the edges. However, a similar vehicle was found to damage the crop in a field with headlands while turning or maneuvering into another plot.

**Figure 5:**
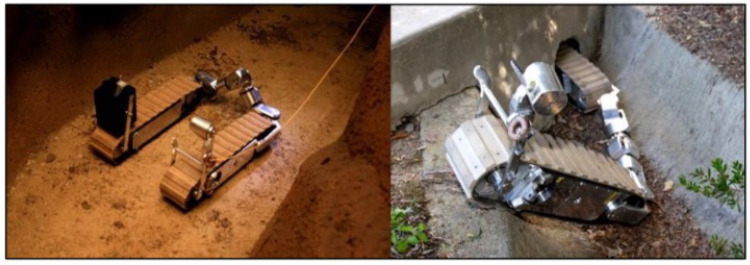
Guardian S robot (left-tank configuration and right-snake configuration) [25].

The survey also reveals that no information is available on reconfigurable vehicles and their application in agriculture. Hence, robotic platforms and vehicle designs in other research domains (military, industry, forestry, etc.) were explored. This survey further introduced the shape-changing robot or reconfigurable vehicle, primarily used for search and rescue operation by performing surveillance. Such robots can maneuver through confined spaces while carrying sensors to survey and map their environment. The Guardian S, shown in Figure 5, can climb metal walls and stairs, and enter tunnels while being controlled via tether or wireless remote. It can operate for 18 h in inspection mode and 4 h of continuous driving. It is very costly, and the base cost is around USD 60,000 [25]. Besides this, shape-changing robots are common in tunnel exploration and have to change shape to overcome obstacles and enter narrow passageways [26]. Reconfiguration is always challenging in shape-transforming robots. Hence, Li et al. (2010) proposed a dynamic shape-shifting approach for the tracked robots [27].

The conducted survey on mobile vehicles explored the various types of robots and ground vehicles. The vehicle design considerations and characteristics of each vehicle were also studied and analyzed. Many of those vehicles have positive aspects required for the scouting job. Still, none were able to meet the challenge of being cost-effective and minimally invasive for row crops. The Rowbot and BoniRob need a different planting strategy or running over crops to complete the scouting in a conventionally planted field. The Guardian S can scout a field without causing damage, but it has low run time, which would require large fleet, increasing the fleet cost. An economical solution is desired to integrate into existing farming practices to complete minimally invasive scouting.

## 3. Materials and Methods

### 3.1. RGV Design Considerations

#### 3.1.1. Crop Spacing

In row crops, plant spacing is determined by plant population. The corn plant population can vary from 12,000–36,000 plants/acre [28], which results in individual plant spacing of 5 cm to 15 cm, respectively. Besides this, planter configuration can also influence the plant spacing, and planter inefficiency further decreases or increases the plant spacing [29]. Corn plants are not consistently or precisely spaced 14–15 cm apart, and the spacing can vary in the actual field. The row-to-row spacing in corn ranges from 75 to 100 cm, which is fixed and consistent throughout the field with headlands. Understanding the basic information on corn plant spacing is crucial for deciding a vehicle dimension, design, and sensor selection. The vehicle should fit in standard row crop spacing and navigate without damaging the crop. Additionally, the vehicle navigating the corn field does not have to be complex or fully aware of every single point in space.

#### 3.1.2. Crop Growth Stage

The pest attack or severity can vary in different crop growth stages. Particularly for corn, a vehicle must scout a field between the growth stages of V3 (emergence of the third leaf) to R1 (silking stage). During these growth stages, various insects or pests can cause severe crop damage [28].

#### 3.1.3. Obstacle Avoidance

The vehicle must navigate through the obstacles such as pivot ruts, washed-out gullies, large exposed rock formations, lodge plants, and other objects. In addition to avoiding obstacles, the vehicle needs to be minimally invasive while traveling the field and through headlands, allowing it to conserve energy and avoid damaging the crop.

#### 3.1.4. Cost-Effective

Scouting and monitoring a large field will require a fleet of vehicles communicating with each other and passing information. Hence, the vehicle design needs to be cost-effective and an economical solution for producers. Besides this, it should be modular and scalable. The cost of the robots can be offset by their ability to adapt to other monitoring tasks.

#### 3.1.5. Adopt to Current Farming Practices

**Figure 6:**
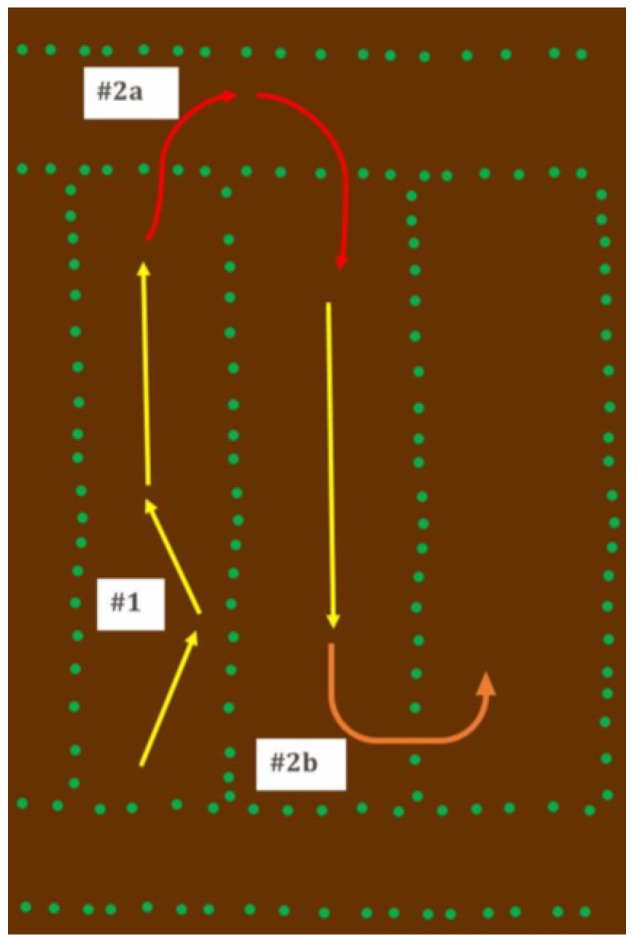
Depiction of maneuvering methods: #1 inter-row; #2 intra row.

A ground vehicle can maneuver in two ways for field scouting: (1) intra-row and (2) inter-row navigation. In inter-row navigation, a vehicle travels between two crop rows, following straight or contoured rows depicted by the yellow arrows in Figure 6. In intra-row navigation, a robotic vehicle moves from one row to another. Intra-row navigation takes place in two ways: #2a, red arrows, exiting the row by moving into the headlands and then entering into the next row, or by #2b, orange arrows, moving directly into the next row by moving between plants. In conventional corn planting practices, non-reconfigurable vehicle design runs down valuable crops when the vehicle travels from one row to the next. Therefore, the developed vehicle must be reconfigurable to avoid crop damage.

### 3.2. RGV Conceptual Design

A primary goal of the RGV is to traverse through inter- and intra-row corn crops with minimal crop damage. After assessing the RGV design consideration (Section 3.1) and many other vehicle designs, the design configuration that best serves the above goal was selected.

**Figure 7:**
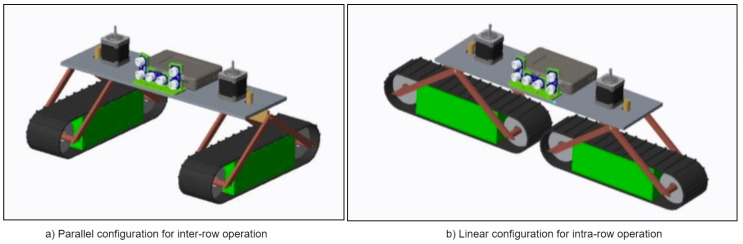
RGV conceptual drawing, (**a**) parallel configuration, (**b**) linear configuration.

A two-track design with a single supporting main frame was selected, as shown in Figure 7. A crawler design was selected for tracks that prioritize stability and maneuverability. The main frame supports the following components: (1) two tracks, (2) two individual track motors for shape-changing, (3) micro-controller (myRio), (4) instrumentation setup, and (5) necessary sensors. Two motors mounted on the main frame turn the track assembly during the shape-changing operation. The RGV will operate in two configurations: (1) parallel configuration (tracks) for traveling inter-rows (Figure 7a) and (2) linear configuration for intra-row (Figure 7b). The parallel configuration resembles the shape of the letter “H”, and the dimension of the RGV was 508 mm (width) × 355.6 mm (length). In the linear configuration, both tracks would rotate to become co-linear with the mainframe, and the dimension of the RGV was 125 mm (width) × 762 mm (length). The linear configuration allows the RGV to travel between two corn plants spaced at 150 mm or through any obstacle with an opening as narrow as 140 mm. Additionally, this configuration reduces the width of the RGV (including the frame and the tracks) to less than 152.4 mm; hence, the RGV can easily maneuver through corn field with high plant population. Besides this, the linear configuration allows the robotic vehicle to move over long ruts or gullies.

### 3.3. Concept of Corn Row Detection

**Figure 8:**
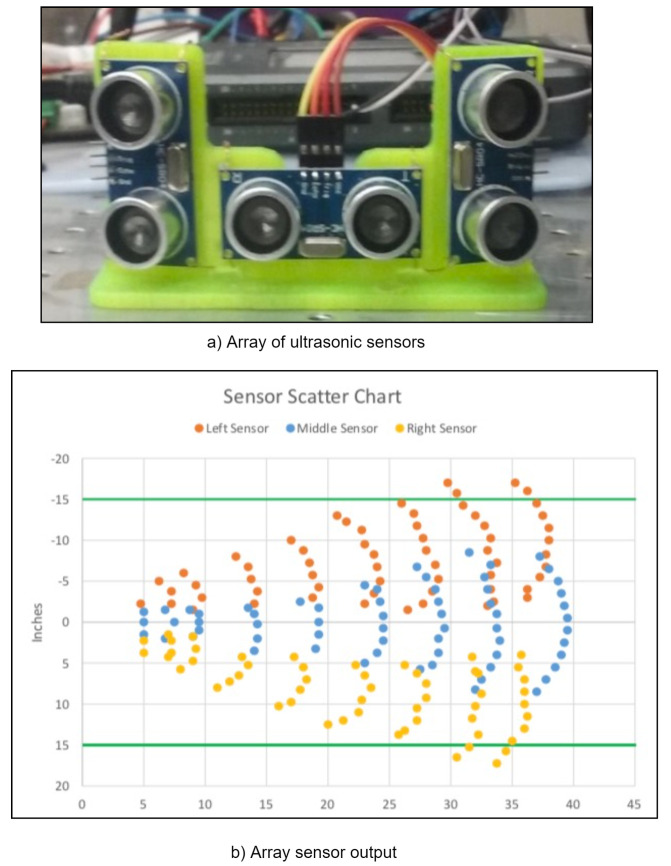
A concept of corn row distance measurement or detection, (**a**) Ultrasonic sensor array, (**b**) Sensor array output.

Corn stalk detection is challenging, and the literature has shown that ultrasonic sensors can detect obstacles in an agricultural setting [30]. However, to the best of our knowledge, there was no research on the application of ultrasonic sensors for navigation, specifically in a corn field. This study used low-cost ultrasonic sensors (USD 2.50/sensor) to detect the corn stalk. An ultrasonic distance sensor (HC-SR04, Sparkfun Inc., Niwot, CO, USA) was selected, which provides a 2 cm to 400 cm non-contact measurement with an accuracy of 3 mm. The sensors have a 30° spread pattern from the sensor’s location. Therefore, an array of three ultrasonic sensors was created, as shown in Figure 8a. The array was fitted on the RGV chassis, which measures the distance from the obstacles or corn at the vehicle’s left, center, and right. In order to avoid obstacles or corn rows, it is essential to find the ultrasonic sensor range. Therefore, a wooden dowel rod that simulates the corn stalk was used to find the sensor range. Each sensor range or output is presented in Figure 8b, and each dot confirms that the sensor sensed the dowel, which provides feedback on the distance from the fixed line. Figure 8b shows the conical shape spread pattern of ultrasonic sensors mounted on the sensor array. The preliminary results suggest that the sensor array provides feedback on corn row position in the field and can be easily integrated into the RGV for its autonomous navigation.

### 3.4. RGV Construction and Fabrication

While developing the RGV, modularity was given priority since the vehicle should be assembled quickly and mass-produced or scaled in quick time.

#### 3.4.1. Track Assembly

**Figure 9:**
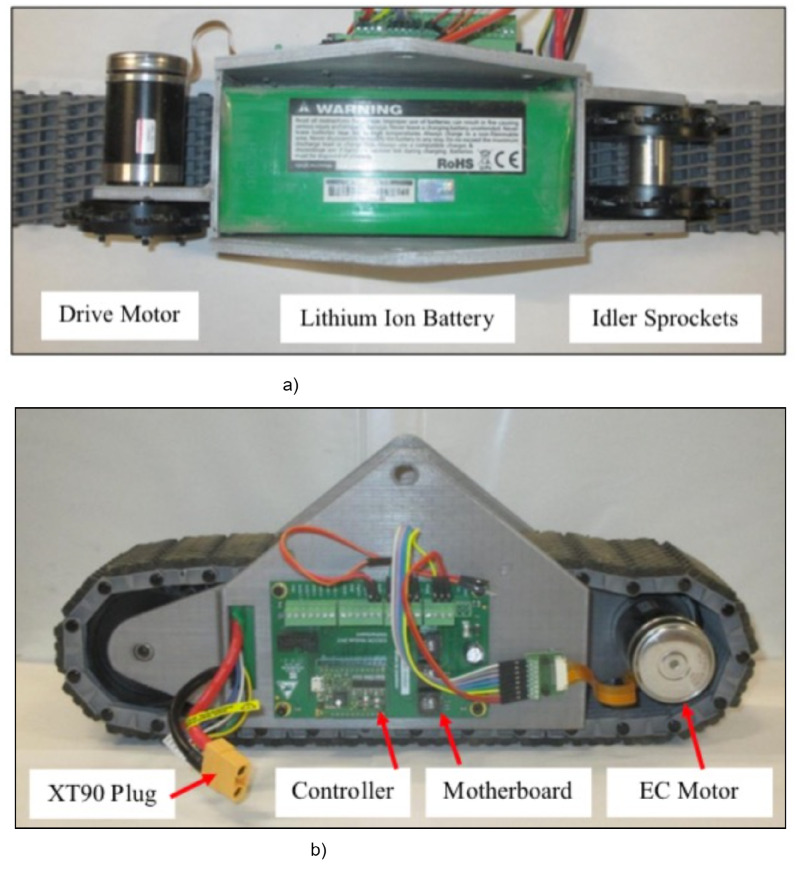
RGV track assembly: (**a**) track assembly showing inside view, (**b**) complete track assembly.

*Tracks:* A rugged polypropylene rubber track (MTS track, Lynxmotion, Robotshop Inc., Swanton, VT, USA) having a width of 76.2 mm was selected. It was a modular track made up of interconnected links and suitable for off-road mobility with good stability. A nine-tooth double-row sprocket (MTS, Lynxmotion, Robotshop Inc., Swanton, VT, USA) having a diameter of 95.2 mm was selected. Each track was fitted with one drive sprocket and two passive idler sprockets, as shown in Figure 9a. The total length of the single track was 368 mm.

*Track motor and controller:* Each track was driven by a 12 V DC motor coupled with a planetary gearhead (Maxon Motor Ag, Sachseln, Switzerland). It provides a maximum continuous torque of 1.42 Nm, and the no-load speed was 167 rev/min. The motor has an in-built encoder for the position feedback. A servo controller (ESCON, Maxon Motor Ag, Switzerland) controls the speed of the track motor. The track motor shaft was coupled to the track drive sprocket (Figure 9a).

*Battery:* Each track was powered with a separate battery. A 22.2 V lithium-ion battery with 16 Ah capacity was used. The battery was fitted inside the track assembly, as shown in Figure 9a. This arrangement offers a compact design and provides a low center of gravity.

**Figure 10:**
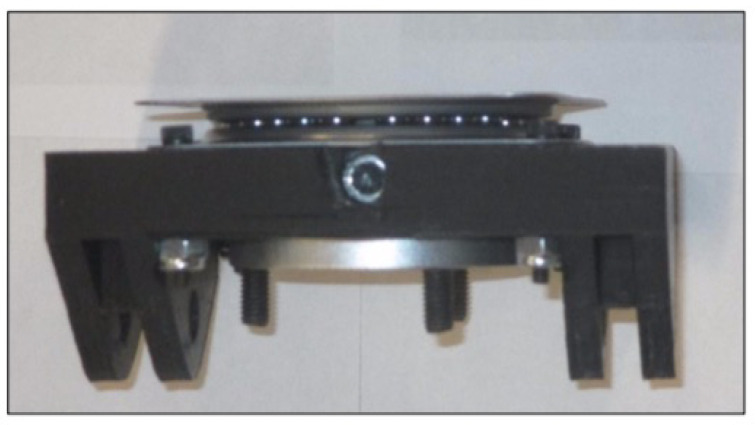
Hinge mount with turntable ball bearing.

*Track frame*: As shown in Figure 9b, a 3D-printed frame was developed to support the geared motor, battery, and sprockets (drive and idler). The track frame was connected to the main frame with the help of a 3D-printed hinge mount (dimension: 107.95 mm × 82.55 mm), as shown in Figure 10, which allows the track frame two degrees of freedom: (1) 360${}^{\circ }$ rotation around the Z-axis and (2) 15${}^{\circ }$ rotation around the Y-axis (lateral). A hinge mount was further connected to a turntable bearing, allowing the rotation in the Z-axis. A turntable bearing provides swiveling action.

#### 3.4.2. Main Frame

The main frame of the RGV was made of aluminum material, and the dimension was 100 mm (width) × 500 mm (length). Both the track frames were connected to the main frame with the help of a hinge mount. The main frame houses the (1) two shape-changing motors (turret motors), (2) a micro-controller, and (3) a sensor package. Both the shape-changing motors were directly connected to the other end of the turntable bearing (attached to the hinge mount) separately.

*Shape-changing (turret) motors:* The turret motors were mounted at the top side of the main frame, and its drive shaft was connected to one end of the turntable bearing (attached to the hinge mount). A turret motor moves the entire track assembly, requiring a high-torque geared motor. Therefore, a 12 V DC geared motor (Lynxmotion, Robotshop Inc., Swanton, VT, USA) having 3 Nm torque and a speed of 10 rev/min was selected. The motor was fitted with an inbuilt hall effect encoder for position feedback. A turret motor’s speed was controlled by a dual H-bridge motor controller (L298, Robotshop Inc., Swanton, VT, USA) which allows a bidirectional rotation. The turret motor rotates the tracks 360${}^{\circ }$, allowing the RGV’s shape to change from a parallel configuration to the linear configuration.

#### 3.4.3. Prototype Details

**Figure 11:**
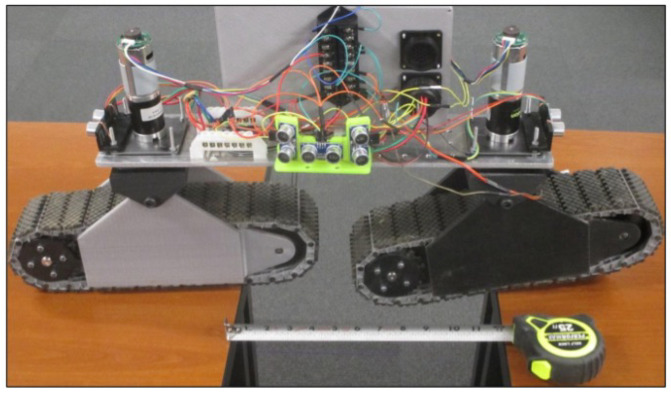
Developed prototype RGV in linear configuration. Note: Prototype was showing the permissible gap to cross pivot ruts.

The fabricated prototype is shown in Figure 11. The dimensions of the final prototype were (1) 508 mm (width) × 355.6 mm (length) in the parallel configuration and (2) 125 mm (width) × 762 mm (length) in linear configuration. The weight of the individual track was 3.4 kg, and the total weight of the RGV was 11.34 kg. The tracks were 76.2 mm wide and 273.05 mm long, which results in a total contact area of 0.021 m${}^{2}$. The maximum speed of the RGV was 1.6 km/h.

### 3.5. RGV Controls and Software

A micro-controller, myRio (National Instruments, Austin, TX, USA), was responsible for the RGV operation and controls. It requires a programming in the LabView environment. The micro-controller controls the speed of track motors and turret motor through the motor controller. The ultrasonic sensor array output was provided to the myRio as distance feedback. The RGV can operate both in autonomous mode and remote operation mode. A remote controller (Optic 5, HiTEC, San Diego, CA, USA) was used for teleoperation, which communicated with the micro-controller (myRio).

## 4. Results and Discussion

Initially, the RGV was tested in a laboratory setup for its functionality. The field trials were conducted in an actual corn field. The RGV scouting ability was assessed during the field test, and power consumption was recorded under different field conditions.

### 4.1. Laboratory Test

**Figure 12:**
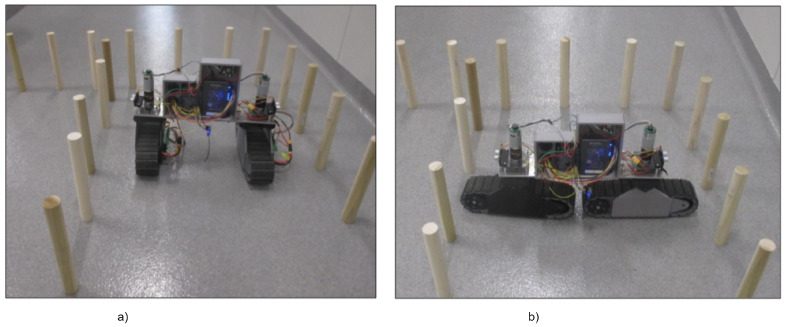
Laboratory test setup of RGV: (**a**) parallel configuration, (**b**) linear configuration.

In the laboratory setup, a corn row was simulated with wooden dowels, allowing different corn spacing for the RGV navigation which included (1) inter-row, (2) intra-row, and (3) a combination of inter- and intra-row navigation. Figure 12 shows the RGV navigation in a laboratory setup, and the maneuvering operation in a laboratory setup includes (1) entering straight rows, (2) following the contoured rows, and (3) navigating from one row to the next. The laboratory simulation showed that the prototype was able to complete the necessary maneuvers to move between simulated rows and plants. In a corn field irrigated with a central pivot system, pivot wheel ruts are very common, and crossing these wheel ruts is important for scout vehicle. The pivot wheel rut was simulated by placing two tables 304 mm apart and driving the prototype across, as shown in Figure 11. The RGV was unable to navigate through pivot ruts in a parallel configuration. However, the RGV’s linear configuration successfully navigated the simulated pivot wheel rut and can cross gaps of 304 mm or smaller. The laboratory-simulated pivot rut was a direct drop off the edge and would be a little different from an actual pivot track rut, which would be round at the edge.

### 4.2. Field Trials

**Figure 13:**
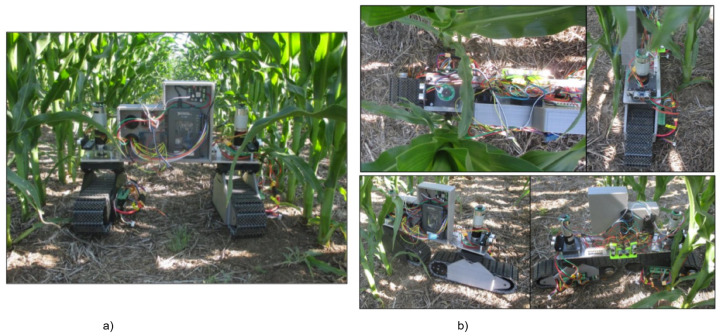
Field trails of RGV: (**a**) parallel configuration, (**b**) linear configuration.

The RGV’s field trials were conducted at Agronomy—NorthFarm, KSU, Manhattan, Kansas. The field had an approximate population of 30,000 plants per acre, and the corn growth stages were V10 and after emergence. The RGV was teleoperated during the field test. The RGV tracks provide sufficient traction for off-road mobility and stable operation. The inter-row navigation was successful, and the RGV had enough space to make turns and make corrections while facing obstacles. The robot successfully reconfigured into the linear configuration for its intra-row maneuver, as shown in Figure 13. In future, attempts will be made to automate the RGV operation with the help of an ultrasonic sensor and additional sensor package, which will guide the RGV through the corn field [31].

#### RGV Power Consumption

The power consumption of the RGV was assessed on the various terrain conditions and is shown in Table 1. The power consumption analysis determines the RGV’s field capacity (coverage range), run time, and recharge frequency. The test length was 50 m, and the RGV was operated on concrete floor, corn, grass, and wheat stubble field. The RGV was driven at maximum speed during the test, and voltage and currents were recorded. The power consumption of the RGV ranges between 45 to 72 W, and it can operate for more than 4 h. However, the power consumption was higher for the wheat stubble field since the stubble created resistance. The Li-Po batteries are recommended for up to 80% discharge capacity [32]; hence, 80% discharge time was calculated for the RGV.

## 5. Conclusions

This paper proposed a reconfigurable scout vehicle for corn crop navigation. Initially, we reviewed the currently available mobile scout vehicles or robots in agricultural and other engineering domains, including search and rescue operation, tunnel exploration, and military operation. The challenges in design, construction, and adoption were identified. The critical findings or successful design aspects were studied, analyzed, and further considered for the proposed vehicle design. A reconfigurable ground vehicle (RGV) was designed and fabricated, and the developed prototype was tested in the laboratory and actual field environment. Moreover, the study also implemented the concept of corn row detection by utilizing an array of low-cost ultrasonic sensors. The following conclusions can be drawn from this study.

1. This research presents a vehicle design aspect for row crop navigation, including intra-row, inter-row, and around-obstacles navigation. The configuration change allows the RGV to maneuver in corn rows and between plants.
2. The developed prototype successfully traverses the corn field and can carry multiple sensor packages.
3. A low-cost ultrasonic sensor proved a robust solution for avoiding obstacles and guiding the robot through the corn stalks. The ultrasonic sensors can be further implemented in the RGV’s autonomous operation by providing obstacle feedback and correcting the RGV’s position or heading.
4. The power consumption of the RGV ranges between 45 to 72 W and it can operate for more than 4 h.
5. The developed RGV is modular, scalable, and can be mass-produced quickly. A fleet of these RGVs would empower the farmers to make meaningful and timely decisions for their cropping system.

This paper proposed a novel and innovative robotic-based approach to monitoring and collecting crop data at a sub-canopy level. The developed robotic platform (RGV) is an excellent alternative to current manual scout methods. For large-scale fields, a swarm of multiple RGVs can be deployed, which would collect and share the real-time information (on crops) with the growers, allowing farmers to make better management decisions. The proposed vehicles provide information on the pest hotspots, pressure, and severity level, allowing targeted site-specific pesticide application, and reducing pesticide application compared to the entire field application method. The site-specific pesticide application would save the chemical inputs, reducing the total production costs and reducing the unsustainable chemical load in environments.

Future research includes adopting the RGV concept in other row crops, including sorghum, soybeans, and sunflowers. For the autonomous operation of the RGV, a fusion of multiple sensors would be tested which includes the LiDAR, GPS, camera, and precise ultrasonic sensor. Additional future work would focus on delivering the concept of the RGV fleet to producers, farmers, and stakeholders for crop scouting. In addition, advanced artificial intelligence based on a computer vision approach would be implemented to identify insect-pest hotspots and severity levels. This fleet would solve the multiple challenges related to insect–pest identification, geolocating their hotspots in large fields, and assessing the pest severity, attacks, and damage. The fleet would become an integral component of integrated pest management operation.

## Figures and Tables

**Table 1:** RGV power consumption on various terrains.

Terrain	Voltage, V	Current, A	Power, W	Ideal Operation Time, h	80% Discharge Time, h
Concrete floor	22.3	2.0	44.6	8.0	6.4
Corn field	22.4	2.4	53.8	6.6	5.3
Wheat stubble	22.3	3.2	71.2	5.0	4.0
Grass	22.3	2.7	60.3	5.9	4.7

## Data Availability

Not applicable.
